# Manipulation of an existing crystal form unexpectedly results in interwoven packing networks with pseudo-translational symmetry

**DOI:** 10.1107/S2059798316013504

**Published:** 2016-09-20

**Authors:** Janice M. Reimer, Martin N. Aloise, Harold R. Powell, T. Martin Schmeing

**Affiliations:** a Department of Biochemistry, McGill University, 3649 Promenade Sir William Osler, Montreal, Quebec H3G 0B1, Canada; b 12 Buxton Road, Chingford, London E4 7DP, England

**Keywords:** NRPS, crystal packing, pseudo-translational symmetry, formylation, interwoven networks

## Abstract

Nonribosomal peptide synthetases (NRPSs) are multimodular enzymes that synthesize a myriad of diverse molecules. Tailoring domains have been co-opted into NRPSs to introduce further variety into nonribosomal peptide products. Linear gramicidin synthetase contains a unique formylation-tailoring domain in its initiation module (F-A-PCP). The structure of the F-A di-domain has previously been determined in a crystal form which had large solvent channels and no density for the minor A_sub_ subdomain. An attempt was made to take advantage of this packing by removing the A_sub_ subdomain from the construct (F-A_Δsub_) in order to produce a crystal that could accommodate the PCP domain. In the resulting crystal the original packing network was still present, but a second network with the same packing and almost no contact with the original network took the place of the solvent channels and changed the space group of the crystal.

## Abstract 2

A nonribosomal peptide synthetase di-domain construct was produced using known crystal packing as a guide, and the resulting crystal has an unanticipated packing.

## 1. Introduction   

Nonribosomal peptides (NRPs), which are small molecules that are produced by nonribosomal peptide synthetases (NRPSs), cover an enormous expanse of chemical space and have uses ranging from green chemicals to important antibiotics (Walsh, 2004 ▸). The large diversity in NRPs comes from the ability of NRPSs to use >500 monomers as substrates and to co-synthetically introduce chemical modifications into these substrates.

NRPSs are modular enzymes in which the initiation module contains adenylation (A) and peptidyl-carrier protein (PCP) domains, and the subsequent elongation modules contain condensation (C), A and PCP domains (Weissman, 2015 ▸; Hur *et al.*, 2012 ▸; Drake *et al.*, 2016 ▸; Tanovic *et al.*, 2008 ▸). The A domain includes a large N-terminal subdomain (A_core_) and a small, mobile C-terminal subdomain (A_sub_) (Yonus *et al.*, 2008 ▸). NRPS modules may also include special tailoring domains, which introduce chemical modifications into the NRPs as they are synthesized (Walsh *et al.*, 2001 ▸). The formylation (F) domain in the F-A-PCP initiation module of the linear gramicidin synthetase (LgrA–E) from *Brevibacillus parabrevis* has been shown to N-formylate the first amino acid, valine, in linear gramicidin (Kessler *et al.*, 2004 ▸). This formyl­ation event allows elongation to continue and is also required for the biological activity of linear gramicidin (Schoenafinger *et al.*, 2006 ▸).

Because of their modular nature and vast substrate pool, NRPSs have been the focus of bioengineering experiments to produce small molecules with novel or improved activities. We aimed to generate structural insight into how LgrA incorporates an F domain into its initiation module and what adaptations the various domains of the NRPS have to undergo to allow a tailoring domain to function within its system. This information could guide future bioengineering endeavours focused on incorporating formylation domains into non­cognate NRPS systems.

We determined the structure of the F-A di-domain in a crystal form that had an apparent solvent content of 82% and had no density for the A_sub_ subdomain (Reimer *et al.*, 2016 ▸). We reasoned that crystallizing F-A_core_ (without A_sub_) would generate crystals with large empty solvent channels that would allow co-crystallization or soaking experiments with the small PCP domain, in order to observe the interaction between the F and PCP domains. However, the removal of the A_sub_ subdomain resulted in a crystal featuring unexpected packing, with a second, identical but largely independent, packing network interwoven with the original network.

## 2. Materials and methods   

### 2.1. Macromolecule production   

The F-A di-domain construct was cloned, expressed, purified and crystallized as described previously (Reimer *et al.*, 2016 ▸). Using site-directed mutagenesis, the F-A_Δsub_ construct was created by introducing two stop codons directly before the A_sub_ sequence in the F-A expression plasmid. F-A_Δsub_ expression and purification was identical to that of F-A with the following additional purification step: F-A_sub_ in a buffer consisting of 1 *M* ammonium sulfate, 25 m*M* Tris–HCl pH 7.5, 2 m*M* β-mercaptoethanol (β-Me) was loaded onto an equilibrated 5 ml HiTrap Phenyl HP column (GE Healthcare) and eluted using a gradient to 25 m*M* Tris–HCl pH 7.5, 2 m*M* β-Me over 30 ml. Fractions containing F-A_Δsub_ were pooled and buffer-exchanged on a HiLoad 16/60 Superdex 200 column (GE Healthcare) equilibrated in 25 m*M* Tris pH 7.5, 150 m*M* NaCl, 2 m*M* β-Me. Macromolecule-production information is given in Table 1 ▸.

### 2.2. Crystallization   

F-A_Δsub_ (10 mg ml^−1^) was crystallized using the vapour-diffusion method in 24-well sitting-drop plates using 2 µl protein solution plus 2 µl of a crystallization solution consisting of 1.3 *M* sodium formate, 0.1 *M* trisodium citrate pH 5.6. Crystallization information is given in Table 2 ▸.

### 2.3. Data collection and processing   

F-A_Δsub_ crystals were prepared for vitrification by replacing the drop solution with mother liquor containing increasing amounts of glycerol [10, 20 and 30%(*v*/*v*)]. Crystals were mounted and vitrified in the nitrogen cryostream at the McGill Centre for Structural Biology for initial screening and were then stored in liquid nitrogen prior to data collection at the Canadian Light Source. Diffraction data were collected on the Canadian Macromolecular Crystallography Facility (CMCF) 08ID-1 beamline using a small-gap undulator and a Rayonix MX-300 CCD detector. Data sets were integrated and scaled using *iMosflm* (Battye *et al.*, 2011 ▸) and *AIMLESS* (Evans & Murshudov, 2013 ▸), respectively, and in parallel using *HKL*-2000 (Otwinowski & Minor, 1997 ▸). Data-collection and processing statistics are given in Table 3 ▸.

### 2.4. Structure solution and refinement   

Initial structure determination of F-A_Δsub_ in space group *P*4_1_2_1_2 was carried out by rigid-body refinement of the existing F-A model (PDB entry 5es6; Reimer *et al.*, 2016 ▸) using *PHENIX* (Adams *et al.*, 2010 ▸). Electron-density maps calculated at 5 Å in *CNS* (Brunger, 2007 ▸) showed the presence of a second F-A_Δsub_ molecule. Molecular replacement was then used to solve the structure in *P*4_1_ with F-A as a model using *PHENIX* (Adams *et al.*, 2010 ▸). The set of reflections used to calculate *R*_free_ in *P*4_1_ was based on the set used in *P*4_1_2_1_2 and expanded to *P*4_1_ using the reflection-file editor in *PHENIX* (Adams *et al.*, 2010 ▸). Iterative model building and subsequent refinements were performed in *Coot* (Emsley *et al.*, 2010 ▸) and *PHENIX* (Adams *et al.*, 2010 ▸). Structure-solution and refinement statistics are given in Table 4 ▸.

## 3. Results and discussion   

We recently solved the structure of the F-A didomain construct to 2.5 Å resolution in a crystal belonging to space group *P*4_1_2_1_2 with an apparent solvent content of 82% (Reimer *et al.*, 2016 ▸). No electron density is visible for residues C-terminal to the four-residue hinge region connecting the A_core_ subdomain to the A_sub_ subdomain (Supplementary Fig. S1) and adjacent, symmety-related molecules blocked previously observed positions of the A_sub_ subdomain (Reger *et al.*, 2008 ▸; Conti *et al.*, 1997 ▸; Yonus *et al.*, 2008 ▸; Tanovic *et al.*, 2008 ▸; Gulick, 2009 ▸; Mitchell *et al.*, 2012 ▸). We had also crystallized F-A-PCP, but initially were only able to obtain poorly diffracting (>9 Å resolution) crystals. Our principal interest in the F-A-PCP construct was to observe the interactions between the F and PCP domains that enable the valine substrate attached to the PCP domain to be formylated. Because the A_sub_ subdomain (∼11 kDa) is not involved in the crystal contacts and was not known to be required for the F domain–PCP domain interaction, we reasoned that removal of the A_sub_ subdomain could lead to a crystal that had large solvent channels, unoccupied by disordered protein regions, that would be suitable for soaking experiments using an entire purified PCP domain (∼8 kDa), for co-crystallization or as the basis for crystals of a future F-A_Δsub_–linker–PCP construct.

F-A_Δsub_ readily formed crystals which diffracted to a resolution of ∼2.8 Å (Table 4 ▸). Diffraction data were indexed and processed in space group *P*4_1_2_1_2 and the resulting electron-density maps showed good density where expected for F-A_Δsub_, but also unexpected electron density for a new, second molecule of F-A_Δsub_. This second copy (‘molecule *B*’) of F-A_Δsub_ occupies the previously empty solvent channels in the F-A lattice. Refinement of a structure containing molecules *A* and *B* in *P*4_1_2_1_2 resulted in molecule *B* being positioned so that it sterically clashed with symmetry-related molecules and gave a higher than expected *R*_free_ of 37%. Further inspection revealed that the electron density in the area of molecule *B* is consistent with two overlapping molecules offset by several Ångstroms, indicating that, as placed, the second molecule did not follow the symmetry of the *P*4_1_2_1_2 space group.

**Figure 1:**
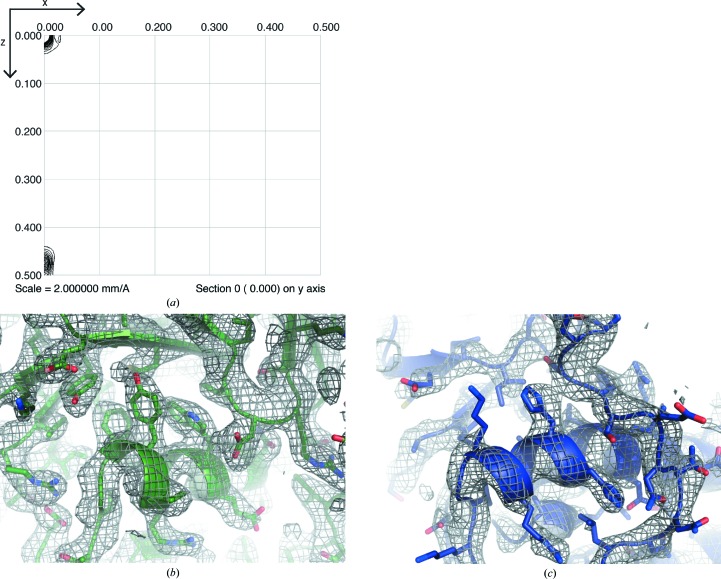
Native Patterson and electron-density maps for F-A_Δsub_. (*a*) The native Patterson map shows a very strong peak (30% of the origin peak) at fractional coordinate position (0, 0, 0.4757) [(0, 0, 66.5 Å) in orthogonal coordinates]. (*b*, *c*) A 2*F*_o_ − *F*_c_ map contoured at 1σ shows density for (*b*) molecule *A* and (*c*) molecule *B*.

We then investigated whether an alternative placement of molecules would follow *P*4_1_2_1_2 symmetry. Molecule *B* of F-A_Δsub_ is related to molecule *A* by noncrystallographic symmetry. Accordingly, the native Patterson map shows a very strong peak (30% of the origin peak) at fractional coordinate position (0, 0, 0.4757) [(0, 0, 66.5 Å) in orthogonal coordinates] (Fig. 1 ▸ *a*), which was not present in native Patterson maps calculated from the data set for the published F-A structure. We could obtain a viable packing arrangement by reassigning the nearest symmetry mate of molecule *A* along *z* as molecule *B* and shifting the coordinates of both molecules *A* and *B* along *z* by *c*/4. Refining these molecules produced a structure with the previously observed clashing resolved, and calculated electron-density maps showed density for a single position of each molecule (Figs. 1 ▸ *a* and 1 ▸ *b*) (rather than the two overlapping molecules offset by several ångströms previously observed for one of the molecules). By keeping one molecule in the position where it appeared in the original structure of the full F-A construct (Reimer *et al.*, 2016 ▸; PDB entry 5es6) and simply fitting a second molecule into the newly appeared density, we had inadvertently misassigned the crystallographic origin and exchanged noncrystallographic symmetry and crystallographic symmetry. This is reminiscent of a recent case of misassigned and then corrected choice of asymmetric unit in crystals of human carbonic anhydrase II (Robbins *et al.*, 2010*a* ▸,*b* ▸).

However, despite the acceptable packing of the molecules when using the proper origin, the *R*_free_ value remained higher than expected (∼33%) after multiple refinement protocols in *PHENIX* (Adams *et al.*, 2010 ▸), *CNS* (Brünger *et al.*, 1998 ▸; Brunger, 2007 ▸) or *REFMAC*5 (Murshudov *et al.*, 2011 ▸). We therefore evaluated the related lower symmetry space groups *P*1, *P*2_1_, *P*2_1_2_1_2_1_ and *P*4_1_, and the true space group was found to be *P*4_1_. The data were reprocessed in *P*4_1_ and molecular replacement was used to place four molecules (*A*, *B*, *C* and *D*) in the asymmetric unit, which was refined to produce the structure reported here, which has an *R*_free_ of 27% and interpretable maps (Figs. 1 ▸ *b* and 1 ▸ *c*). It should be noted that performing the analogous protocol with the other space group related to *P*4_1_2_1_2 by loss of one symmetry element, *P*2_1_2_1_2_1_, led to a worse *R*_free_ of 33%.

Distinguishing the space group as *P*4_1_ instead of *P*4_1_2_1_2 was not facile. The data are processed easily in *P*4_1_2_1_2, with both *phenix.xtriage* (Adams *et al.*, 2010 ▸) and *POINTLESS* (Evans, 2006 ▸) suggesting *P*4_1_2_1_2 as the correct space group. Indeed, inspection of the reflections that should be systematically absent in *P*4_1_2_1_2 but not *P*4_1_ [average *I*/σ(*I*) = 1.2 for (*h*00); *h* = 2*n*] and comparison of the CC_1/2_ and *R*_merge_ values in each space group (*P*4_1_, 0.085; *P*4_1_2_1_2, 0.089; calculated to 3 Å) did not indicate a clear distinction between space groups. Furthermore, *Zanuda* (Lebedev & Isupov, 2014 ▸), which evaluates real-space packing symmetry, indicated that the packing was compatible with both *P*4_1_ and *P*4_1_2_1_2. These results can be rationalized by comparison of the *P*4_1_ and *P*4_1_2_1_2 structures. The positions of the molecules (including symmetry mates) in the *P*4_1_ and *P*4_1_2_1_2 structures can be superimposed well, with the exception of approximately five side chains. However, in *P*4_1_ molecules *A* and *C* are well ordered, as indicated by strong electron density (Fig. 1 ▸ *b*), moderate *B* factors (the average *B* factor of non-H atoms is 64 Å^2^) and good geometry statistics, while molecules *B* and *D* have weaker electron density (Fig. 1 ▸ *c*) and therefore very high *B* factors (average of 130 Å^2^) and poorer geometry statistics. When processed in *P*4_1_2_1_2 (with the correct origin), molecule *A* of *P*4_1_ averages with molecule *D* and molecule *B* with molecule *C*. This combining of more and less ordered molecules in *P*4_1_2_1_2 results in intermediate *B* factors, but the structure cannot be sufficiently well modelled and produced a distinctly higher *R*_free_ (∼33%). Indeed, the same higher *R*_free_ (∼33%) is obtained in *P*4_1_ if the intermediate *B* factors are applied to all molecules. Thus, in *P*4_1_ and not in *P*4_1_2_1_2, the more and less ordered molecules can be treated separately and the model refined to an acceptable *R*_free_ (∼27%). It is not clear why molecules *B* and *D* have higher *B* factors. They may display dynamic disorder, static disorder or may actually be at lower occupancy. If molecules *A* and *C* are present throughout the crystal and molecules *B* and *D* are present substoicheometrically, the lowering of symmetry observed here would be a similar to the lowering of symmetry observed in small-molecule crystals by the stochastic presence of a ‘guest’ in a ‘host’–‘guest’ system (Weisinger-Lewin *et al.*, 1989 ▸). Attempts to refine the occupancy were not fruitful, which is perhaps not surprising given the moderate resolution of the data set and the strong inverse correlation of occupancy and *B* factors.

**Figure 2:**
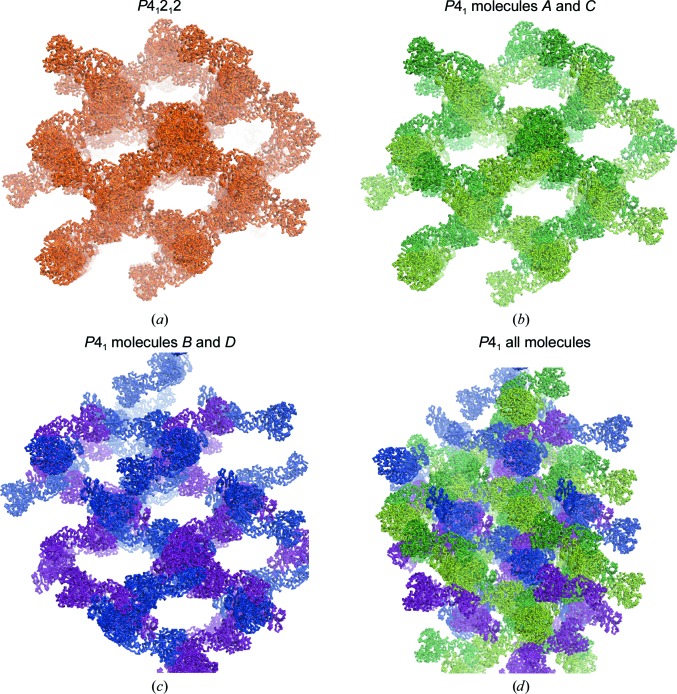
Pseudo-independent packing networks in the F-A_Δsub_ structure. (*a*) The lattice observed in the published crystal structure of the LgrA F-A construct (Reimer *et al.*, 2016 ▸; PDB entry 5es6) in space group *P*4_1_2_1_2. In the new *P*4_1_ crystal, molecules *A* and *C* (*b*) form a packing network essentially identical to that of F-A, and molecules *B* and *D* (*c*) form a separate, translationally related network, which combine to result in the F-­A_Δsub_ lattice (*d*).

**Figure 3:**
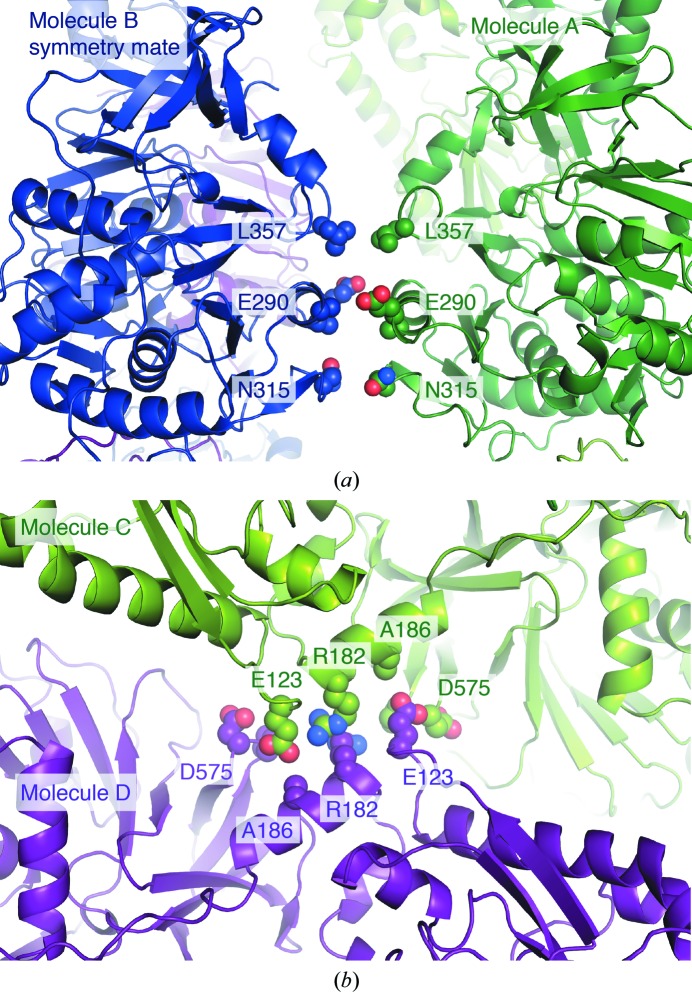
Crystal contacts between the *A*–*C* and *B*–*D* packing networks. Molecule *A* in the F-A_Δsub_ structure uses three residues (*a*) to pack against the same residues in the neighbouring symmetry mate of molecule *B*. Molecule *C* and molecule *D* form a small interaction surface (*b*) by packing antiparallel with the same four residues. See Supplementary Table S1 for lists of buried surface areas.

Molecules *A* and *C* display the same crystal contacts and packing network as observed in the F-A crystal (Fig. 2 ▸). Remarkably, molecules *B* and *D* also form the very same packing arrangement, but this is translated 66.5 Å along the *c* axis into the space where the large solvent channels are in the F-A crystal (Fig. 2 ▸). We find it quite notable that although the *A*–*C* and *B*–*D* networks are practically identical and interwoven, they make very little contact with each other. The only two interaction areas are (i) a small patch of residues around Glu123 and Arg182 of molecule *C*, which interacts with the same residues in molecule *D*, and (ii) a patch around Asn315 of molecule *A* which interacts with the same residues of molecule *B* from the adjacent asymmetric unit (Fig. 3 ▸). These contacts bury only 289 Å^2^ of surface area, which contrasts markedly with the 3028 Å^2^ of surface area that each molecule buries within the *A*–*C* or *B*–*D* packing networks (Supplementary Table S1).

**Figure 4:**
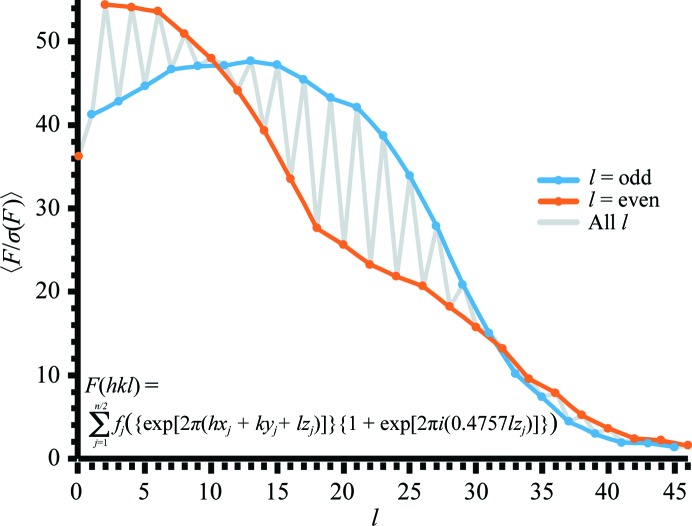
Effect of the pseudo-translational symmetry on amplitudes in the diffraction data. The average *F*/σ(*F*) for the *hkn* zones of reciprocal space plotted as a function of *l*. This shows the effect of the {1 + exp[2π*i*(0.4757*lz_j_*)]} term in the structure-factor equation summed over *n*/2 atoms with the translational symmetry applied (Sundlov & Gulick, 2013 ▸).

The pseudo-translational symmetry (Chook *et al.*, 1998 ▸) displayed by these packing networks has a noticeable effect on the diffraction data (Wang *et al.*, 2005 ▸; Tsai *et al.*, 2009 ▸). In addition to the strong peak in the native Patterson (Fig. 1 ▸ *a*), the pseudo-translational symmetry is also evident from the fluctuations in the average amplitudes in the *l* = even and *l* = odd zones (Fig. 4 ▸; Sundlov & Gulick, 2013 ▸; Tsai *et al.*, 2009 ▸). We note that the intensity patterns in the diffraction data are reminiscent of lattice-translation defects (Wang *et al.*, 2005 ▸; Tsai *et al.*, 2009 ▸; Zhu *et al.*, 2008 ▸). However, we do not observe the characteristic pattern of streakiness (alternating sharp–diffuse reflections or large orientation-specific variations in streakiness) in any of the data sets that we collected from this crystal form. Also, it would be unusual for a lattice-translation defect to result in a packing arrangement that is fully compatible with the nontranslated lattice. It should also be noted that the intensities of reflections did not indicate the presence of twinning in the data, for example using the *L*-test in *phenix.xtriage* (Zwart, 2005*a* ▸,*b* ▸).

Interwoven packing networks and pseudo-translational symmetry are not rare in protein crystals (Sakai *et al.*, 2014 ▸; Ringler & Schulz, 2003 ▸; MacKinnon *et al.*, 2013 ▸; Zwart *et al.*, 2008 ▸; Chook *et al.*, 1998 ▸; Sundlov & Gulick, 2013 ▸). However, we do not know of another case where attempts to manipulate a crystal form led to a different crystal form featuring a duplication and translation of the original packing network.

Our attempts to alter a crystal form to provide more space for the binding of a partner domain had the exact opposite effect. Removal of the A_sub_ subdomain allowed a doubling of the number of protein molecules present in the unit cell, with the new molecules related by translational noncrystallographic symmetry making almost no contact with the original molecules. Unfortunately, the new molecules block all potential access to the active site of the F domain. Therefore, PCP domain soaking or co-crystallization experiments do not seem to hold promise in this crystal form. Fortunately, we were subsequently able to obtain a new crystal form that showed F-A-PCP in the formylation state (Reimer *et al.*, 2016 ▸). Therefore, this doubling did not represent a major setback in our studies, but instead is a benign and unexpected consequence of attempting to alter an existing crystal form.

## Supplementary Material

PDB reference: LgrA initiation module excluding the A_sub_ domain (F-A_Δsub_), 5jnf

Suppporting Information.. DOI: 10.1107/S2059798316013504/yt5098sup1.pdf

## Figures and Tables

**Table 1:** F-A_Δsub_ production information

Source organism	*B. parabrevis*
DNA source	ATCC 8185 (Cedarlane Laboratories)
F-A_Δsub_-S†	TTTCTCGGCCGCCTGGACTAATAAAATTTGGTCAAAATTCGCGGATACC
F-A_Δsub_-AS†	GGTATCCGCGAATTTTGACCAAATTTTATTAGTCCAGGCGGCCGAGAAA
Expression vector	pET-21-derived vector
Expression host	*E. coli* BL21 (DE3)
Complete amino-acid sequence of the construct produced	GAMGRILFLTTFMSKGNKVVRYLESLHHEVVICQEKVHAQSANLQEIDWIVSYAYGYILDKEIVSRFRGRIINLHPSLLPWNKGRDPVFWSVWDETPKGVTIHLIDEHVDTGDILVQEEIAFADEDTLLDCYNKANQAIEELFIREWENIVHGRIAPYRQTAGGTLHFKADRDFYKNLNMTTVRELLALKRLCAEPKRGEKPIDKTFHQLFEQQVEMTPDHVAVVDRGQSLTYKQLNERANQLAHHLRGKGVKPDDQVAIMLDKSLDMIVSILAVMKAGGAYVPIDPDYPGERIAYMLADSSAAILLTNALHEEKANGACDIIDVHDPDSYSENTNNLPHVNRPDDLVYVMYTSGSTGLAKGVMIEHHNLVNFCEWYRPYFGVTPADKALVYSSFSFDGSALDIFTHLLAGAALHIVPSERKYDLDALNDYCNQEGITISYLPTGAAEQFMQMDNQSFRVVITGGDVLKKIERNGTYKLYNGYGPTECTIMVTMFEVDKPYANIPIGKPIDRTRILILDEALALQPIGVAGELFIVGEGLGRGYLNRPELTAEKFIVHPQTGERMYRTGDRARFLPDGNIEFLGRLD

† The underlined sequence denotes the stop codon inserted between the A_core_ and A_sub_ sequences.

**Table 2:** F-A_Δsub_ crystallization

Method	Vapour diffusion (sitting drop)
Plate type	24-well plate
Temperature (K)	298
Protein concentration (mg ml^−1^)	10
Buffer composition of protein solution	150 m*M* NaCl, 25 m*M* Tris–HCl pH 7.5, 2 m*M* β-Me
Composition of reservoir solution	1.3 *M* sodium formate, 0.1 *M* trisodium citrate pH 5.6
Volume and ratio of drop	2 µl + 2 µl
Volume of reservoir (µl)	500

**Table 3:** Data collection and processing Values in parentheses are for the outer shell. The F-A data set was cut at *I*/σ(*I*) > 2.

Diffraction source	Canadian Light Source beamline 08ID-1
Wavelength (Å)	0.979
Temperature (K)	99
Detector	Rayonix MX-300 CCD
Crystal-to-detector distance (mm)	375
Rotation range per image (°)	0.4
Total rotation range (°)	179.6
Exposure time per image (s)	1
Space group	*P*4_1_
*a*, *b*, *c* (Å)	161.3, 161.3, 139.9
α, β, γ (°)	90.0, 90.0, 90.0
Resolution range (Å)	88.41–2.55 (2.60–2.55)
Total No. of reflections	867930 (36367)
No. of unique reflections	116252 (5760)
Completeness (%)	100 (100)
Multiplicity	7.5 (6.3)
〈*I*/σ(*I*)〉	8.0 (0.8)
*R*_r.i.m._	0.083 (1.317)
Overall *B* factor from Wilson plot (Å^2^)	59.1

**Table 4:** F-A_Δsub_ structure solution and refinement Values in parentheses are for the outer shell.

Resolution range (Å)	47.92–2.75 (2.84–2.75)
Completeness (%)	99.23 (99.0)
σ Cutoff	2.0
No. of reflections, working set	88204
No. of reflections, test set	4416
Final *R*_work_ (%)	23.96 (40.81)
Final *R*_free_ (%)	27.23 (38.84)
No. of non-H atoms	
Protein	18470
Ion	15
Water	180
Total	18665
R.m.s. deviations	
Bonds (Å)	0.004
Angles (°)	0.640
Average *B* factors (Å^2^)	
Protein	97.24
Ion	64.70
Water	61.27
Ramachandran plot	
Most favoured (%)	97.15
Allowed (%)	2.63
PDB code	5jnf
