# Supplementary material for: Manipulation of an existing crystal form unexpectedly results in interwoven packing networks with pseudo-translational symmetry
Source: Acta Crystallogr D Struct Biol. 2016 Sep 20;72(Pt 10):1130–6. doi: 10.1107/S2059798316013504 (PMC5053139; doi:10.1107/S2059798316013504)
Supplement: Supplementary file 1 [file d-72-01130-sup1.pdf]

STRUCTURAL BIOLOGY

Volume 72 (2016)

Supporting information for article:

Manipulation of an existing crystal form unexpectedly results in interwoven packing networks with pseudo-translational symmetry

Janice M. Reimer, Martin N. Aloise, Harold R. Powell and T. Martin Schmeing

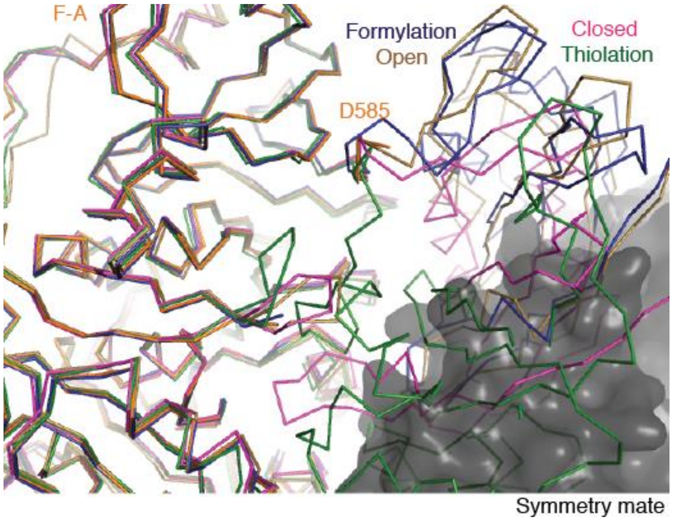

Figure S1 A_{sub} subdomain disorder in the published crystal structure of the LgrA F-A construct. Only the A_{core} subdomain is well-ordered in the published structure of the full FA construct ((Reimer *et al.*, 2016); PDB ID 5ES6) (shown in orange), allowing modelling up to residue D585 of the hinge region. A symmetry mate (shown as a grey semi-transparent surface) prevents the A_{sub} subdomain from adopting any of its known functional positions. (Formylation: PDB ID 5ES9 chain A – shown in blue; Open and closed: PDB ID 5ES5 chain A and B – shown in magenta, respectively; Thiolation: PDB ID 5ES8 – shown in green).

Table S1 Surface area buried by each crystal contact.

**Contact partners**	**Buried surface area per molecule**	**Inter or intra packing network**
molecule A - molecule C	1628 Å^2	intra A - C network contact
molecule A - molecule C symmetry mate 1	598 Å^2	intra A - C network contact
molecule A - molecule C symmetry mate 2	562 Å^2	intra A - C network contact
molecule A - molecule C symmetry mate 3	240 Å^2	intra A - C network contact
molecule C - molecule B	206 Å^2	inter AC - BD network contact
molecule A - molecule B symmetry mate	83 Å^2	inter AC - BD network contact
sum; intra-packing network contacts	3028 Å^2	intra A - C network contact
sum; inter-packing network contacts	289 Å^2	inter AC - BD network contact
